# Influence of Socio-Economic Status on Psychopathology in Ecuadorian Children

**DOI:** 10.3389/fpsyt.2020.00043

**Published:** 2020-02-14

**Authors:** María Nieves Pérez-Marfil, Manuel Fernández-Alcántara, Ahmed F. Fasfous, Carlos Burneo-Garcés, Miguel Pérez-García, Francisco Cruz-Quintana

**Affiliations:** ^1^ Mind, Brain and Behavior Research Center (CIMCYC-UGR), University of Granada, Granada, Spain; ^2^ Department of Health Psychology, University of Alicante, Alicante, Spain; ^3^ Department of Social Sciences, Bethlehem University, Bethlehem, Palestine; ^4^ Carrera de Derecho, Universidad de Otavalo, Otavalo, Ecuador

**Keywords:** socioeconomic status, psychopathology, internalizing, externalizing, anthropometric measures, child behavior

## Abstract

The socioeconomic status (SES) of parents has been reported to have a crucial impact on emotional competence in childhood. However, studies have largely been carried out in developed countries and in children in a specific age range, and it is not clear whether the effect of the SES of parents varies by age. The objective of this study was to investigate the psychopathological profile (including externalizing and internalizing problems) of children aged 7, 9, and 11 years old with low SES in a developing country (Ecuador). The study included 274 children (139 boys and 135 girls), who were divided between medium-SES (n = 133) and low-SES (n = 141) groups. Data were gathered on socioeconomic and anthropometric variables of the children, and the parents completed the Child Behavior Check-List (CBCL). In comparison to the medium-SES group, children in the low-SES group obtained higher scores for internalizing and externalizing symptoms and for total problems, and they obtained lower scores for social competence skills. The housing risk index and school competence were the two main predictors of internalizing and externalizing problems in this population.

## Introduction

Neurodevelopment is a dynamic inter-relationship among genetic, cerebral, cognitive, emotional, and behavioral processes over life (1). Emotional competence and skills can be influenced by nutritional, infectious, and toxic factors and by upbringing practices and patterns (2), as well as by the socio-economic status (SES) of parents (3–6).

SES consists of numerous factors, including family income, parental education and occupation, psychological and physical health status, physical conditions at home, upbringing practices, stressful situations suffered by child and/or parents, physical or psychological abuse, and nutritional status (7–11). It has been reported that low SES can be a risk factor for inadequate socio-emotional development and can increase vulnerability to development problems (12–14). Various theoretic models have been proposed for the impact of SES on neurodevelopment, considering different explanatory variables. Thus, a low SES has been associated with: external factors, related to the environment with which the child interacts; and internal factors, more related to interactions with parental figures.

External factors are considered in the model by Evans, among others, in which a low SES is associated with greater exposure to environmental risk, which in turn increases the risk of physical and psychological disorders. The review by Evans and Kantrowitz (15) identified a large number of environmental stressors that can indirectly influence the physical and emotional health of children living in poverty, including: lower air quality, larger presence of environmental toxins, higher ambient noise, greater residential crowding, lower housing quality, and worse educational facilities.

Numerous theoretic models have focused on the influence of low SES and poverty on the interaction between parents and children, acknowledged to be a crucial factor (16, 17). Although no unified model has been published that considers all stressors associated with a low SES, certain key factors have been identified (18), including parental investment, parental practices, levels of chronic stress, and coping (8, 19, 20). Parental investment refers to the quality of cognitive stimulation at home, including the availability of books, electronic devices, and toys and the amount of time spent watching television. Parental practices refer to the interaction or involvement of parents in their children's care. It has been suggested that these practices are characterized by greater hostility and conflict in a low SES environment, with less care and support of the children and inconsistent punishment and reward patterns, favoring the development of internalizing and externalizing problems in the children (21). Finally, children with low SES have been found to have lower skills to deal with these stressors, which can accumulate over time and interact with each other, as well greater difficulty in controlling their emptions (19).

Middle childhood (7–11 years) appears to be a crucial stage for the regulation of emotions and for the onset of psychopathologic disorders It is a time when social skills develop, sexual differences are maximized, and important interactions between environmental and genetic factors can be observed (22). In this pre-adolescent stage, there is a marked development of executive function and self-regulatory capacities (23) but also a greater risk of psychopathologic problems. It has been reported that psychopathologies appearing during infancy start to increase at the age of 5–6 years and reach their main peak in middle childhood (24) and that the likelihood of such problems at this stage is increased in a low SES environment (25).

Recent studies on the association between a low SES and psychopathologic aspects showed that children from poorer backgrounds experience greater difficulties in controlling impulses and delaying gratification (26). It has also been observed that the accumulation of multiple risks (e.g., deficits in education, mistreatment, or lack of parental attention) during early infancy can predict a lower capacity for emotional self-regulation, with difficulties in controlling emotions and in co-operating with peers and independent play (10, 27–29).

Special interest has been shown in the relationship between SES and the development of mental health problems in children and adolescents, specifically internalizing and externalizing problems, (4, 25). It is estimated that around 20% of children and adolescents worldwide have mental health problems (30), which are considered to be two- to three-fold more prevalent in children of families with low SES (31). The influence of this condition on the short- and long-term development of mental health problems is considered to be more marked during the first months of life (31). However, it has also been found that children and adolescents growing up in a low SES environment have more internalizing (e.g., anxiety and depression) and externalizing (e.g., aggressiveness, opposition, and hyperactivity) symptoms, although findings have not been consistent (4).

There have been attempts to identify the mechanisms underlying the effect of SES on the mental health of children. Bøe and colleagues (4) reported that the emotional wellbeing of parents and their upbringing practices may act as mediator of the interaction between SES and mental health problems in children. They observed that the family economy and the educational level of parents affected the mental health of children in different ways. Thus, the family economy was related to the presence of externalizing problems through the emotional wellbeing of parents and upbringing practices, while the educational level of the mother was related to externalizing problems through negative disciplinary methods. In the case of internalizing problems, direct and indirect associations were found with the family economy, mediated by maternal emotional wellbeing and upbringing practices (4).

However, research to date has been limited. Despite the clear relationship between SES and mental health problems in children, few data are available on the prevalence of these problems, on the most frequent types of disorder, or on possible comorbidities (4, 31). One objective of developmental psychopathology has been to analyze the heterogeneity of symptoms presented by children with behavior problems, with the aim of identifying subgroups with similar difficulties for specific treatment approaches (14). However, there has been inadequate consideration of the cultural setting or the country's level of development, with most research being conducted in developed nations (11, 32). In addition, most studies have focused more on the economic aspects of SES and less on the possible influence of other important factors, such as the educational level of parents (14). The family economy and educational level of parents may have differential influences on family processes and children's socio-emotional adjustment. The upbringing of children may be directly affected by the educational level of parents and indirectly affected by the family economy *via* effects on parental emotional wellbeing and mental health. Analysis of individual SES components is needed to determine their specific contribution to the socio-emotional adjustment of children. Thus, Merz et al. (25) reported that anxiety and depression levels of children were negatively related to the educational level of parents but were not associated with family income. Finally, the duration of exposure to a low SES environment can determine the likelihood of psychopathologic problems, so that it is important to take the age of children into account. Various reviews have concluded that experiences of poverty during early childhood can have a damaging effect on both cognitive and emotional development (21). In this line, recent studies have described significant interactions between the age of children and SES levels in verbal memory, phonetic fluency, abstract reasoning, and inhibitory control (13).

Limited data are available on the relationship of SES with the development of psychopathologies during middle childhood. Therefore, the aim of this study was to investigate socio-emotional development in children of 7, 9, and 11 years of age exposed to low or medium SES in a city in a developing country (Guayaquil, Ecuador). The study hypothesis was that the children with low SES would have more emotional (externalizing and internalizing) problems in comparison to the children with medium SES.

## Method

### Participants

The study included 274 Spanish-speaking schoolchildren from Guayaquil, the most populous city of Ecuador, divided among 7-year-olds (45 boys, 44 girls), 9-year-olds (45 boys, 46 girls), and 11-year-olds (47 boys, 45 girls). These ages were selected to allow investigation of the relationship between SES and psychopathologic changes throughout middle childhood.

The study population was divided by SES between: a medium-SES group (n = 133) containing 45 7-year-olds (23 boys, 22 girls), 44 9-year-olds (22 boys, 22 girls), and 44 11-year-olds (22 boys, 22 girls); and a low-SES group (n = 141) containing 46 7-year-olds (24 boys, 22 girls), 47 9-year-olds (23 boys, 24 girls), and 48 11-year-olds (25 boys, 23 girls).

### Sampling Procedure

The study was conducted in primary schools in the city, selected to provide a balanced representation of areas with predominantly low-SES or medium-SES populations. Random sampling was conducted among the 7-, 9-, and 11-year-old children registered at each participating school.

The characterization of school catchment areas as low or medium SES and their inclusion in the study was based on multiple factors, including: the private, subsidized, or public funding of the school; basic services in the area; and income and employment levels, among others. Following these criteria, a selection was made of three medium-SES schools (one public, one subsidized, and one private) in the north/center of the city and two low-SES schools (two public schools) in an area at the southern edge of the city “Isla Trinitaria,” surrounded by an inlet of the sea. The population of Isla Trinitaria has considerably increased over the past 20 years, with a major influx of people from other parts of the country, and it currently has around 350,000 inhabitants. It is considered to be one of the poorest zones in the metropolitan area of Guayaquil and did not have access to basic services until 2011, when power lines and a fresh drinking water system were installed, followed by an expansion of the sewage network in 2013. However, major public investment is required in health, education, security, roads, and public transport, among others, before the population of this area can enjoy the same quality of life and opportunities as the majority of the city dwellers (medium-SES).

### Inclusion Criteria

Study inclusion criteria were: a) age of 7, 9, or 11-years at the time of assessment; b) regular attendance at one of the participating schools; c) absence of physical, psychological, or cognitive impairment diagnosed by a specialist or reported by teachers or parents; and d) informed consent signed by parent/guardian. Before evaluation of the selected children, interviews were conducted with their teachers and with their parents/guardians to verify that the above inclusion criteria were met, confirming that none had diagnosed or apparent physical or psychological disorders or evidenced major behavioral problems. The availability of an appropriate room for interviews with the children was also established. Out of the eligible children enrolled in the study, 24 were subsequently excluded due to the withdrawal of consent (n = 4) or because conditions for the assessment were not adequate due to interruptions for school activities (n = 20).

### Instruments

#### Socioeconomic Status

##### Socioeconomic Survey

The questionnaire was developed by the School of Nursing of the University of Guayaquil (33) as part of its Child and Adolescent Care Program and was designed to gather data on the socioeconomic level of the families of children. This questionnaire was administered to the parents/guardians in interviews held at the school of their children (afternoon sessions).This instrument classifies families according to raw scores for maternal level of education level (score range of 1–4) and social class of the head of household (score range of 1–4), and a transformed housing risk index, including house structure, overcrowding, water supply, garbage disposal, toilet availability, and sewage disposal (score range of 1–3). A higher questionnaire score indicates lower socioeconomic level.

#### Anthropometric Measurements

Measurements were taken of the height, weight, and cranial and abdominal circumferences of the children, using: a SECA wall plastic height scale, model 206 (Hamburg, Germany) with measurement range of 0–220 cm; a SECA digital floor scale, model 803 (Hamburg, Germany) with a limit of 150 kg in 100 g increments; and a SECA measuring tape, model 201 (Hamburg, Germany), an ergonomic and flexible band to measure circumferences, with a range of 0–250 cm in 1 mm increments.

#### Psychopathology

##### Child Behavior Checklist in 6- to 18-Year-Olds

The Child Behavior Checklist (CBCL) (34) was used to obtain information from the usual guardians of the children on the children's skills or competences (Social Competence Scale), problematic behaviors (Problems Scale), and Diagnostic and Statistical Manual (DSM)-oriented problems. The social competence items yield scores for three narrow-band scales (activities, social, and school) and one broad-band scale (total social competence). The Problems Scale evaluates eight syndromes: somatic complaints, anxiety, depression, social problems, thought problems, attention problems, rule-breaking behavior, and aggressive behavior. It also allows assessment of two large groups of syndromes: internalizing problems (combining withdrawal, somatic complaints, and anxiety/depression) and externalizing problems (combining rule-breaking behavior and aggressive behavior). The DSM-oriented problems include affective problems, anxiety problems, somatic problems, attention deficit/hyperactivity problems, oppositional defiant problems, conduct problems, sluggish cognitive tempo, obsessive-compulsive problems, and post-traumatic problems. Administration of the CBCL takes 25–30 minutes. Correlation coefficients of .90 were obtained for mean scores between different examiners and between two parental reports separated by 7 days (test-retest reliability). Correlation coefficients for the repeated parental reports were .87 for the Social Competence Scale and.89 for the Problems Scale. The CBCL has been adapted to a wide number of Spanish-speaking countries including Spain, Chile, Mexico, and Puerto Rico (35). In the present study the Spanish version of the CBCL was used (35, 36). To stablish the cut-off scores in the present population, the recommendations of Achenbach and Rescorla (37) were considered.

### Procedure

A team of six trained evaluators carried out the fieldwork during a 4-month period. Interviews and anthropometric measurements of the children were conducted at school during the morning in a room with adequate physical conditions for this purpose. Parents attended a 30-min interview in the afternoon at their children's school to record their socioeconomic data and complete the CBCL. Written consent was obtained from the parents/guardians of the children for their participation in the study, which was approved by the ethical committee of the local University (Ref: A3/042954/11).

### Data Analysis

After descriptive analysis of the data, ANOVAs were conducted with 2x2x3 factorial design considering two SES groups (medium and low), two sex groups (boy and girl), and three age groups (7, 9, and 11 years) as independent variables and CBCL subscales, parental socioeconomic survey subscales, and anthropometric variables as dependent variables, followed by application of the *post-hoc* Bonferroni test. The chi-square test was also applied to evaluate differences among groups in the percentage of clinical problems in each CBCL scale. Finally, linear regression analyses were performed to identify the SES components and social competences with greatest influence on internalizing, externalizing, and total problems of the CBCL. Given the need for multiple comparisons, the Bonferroni correction was applied to reduce the probability of a type I error, establishing the significance threshold at ≤ 0.002 for ANOVAs and ≤ 0.006 for linear regressions. Partial eta-squared was used as effect size measure.

## Results

Before the statistical analyses were performed, the SES classification of participants was tested by considering their maternal education, home risk index, and the social class of head of household. Results confirmed that the classification of the children was appropriate, with the low-SES group scoring significantly higher (i.e., lower SES) for maternal education level [F(1,260) = 249.04, p < .001; partial η^2^ = .522], home risk index [F(1,260) = 104.91, p < .001; partial η^2^ = .290], and social class of head of household [F(1,260) = 256.19, p < .001; partial η^2^ = .502]. No significant differences were observed in these scores as a function of the child's age (see **Table 1**).

**Table 1:** Group, age, and sex differences and interaction on socioeconomic, anthropometric, and nutritional measures.

Measures	Group	7 years		9 years		11 years		*p*	*Post hoc*
	Medium-SES (n = 133) Low-SES (n = 141)	Boy (n = 45) ME (SD)	Girl (n = 44) ME (SD)	Boy (n = 45) ME (SD)	Girl (n = 46) ME (SD)	Boy (n = 47) ME (SD)	Girl (n = 45) ME (SD)		
**Socioeconomic characteristics**									
Maternal education level	Medium Low	1.38 (0.50) 3.43 (1.08)	1.76 (0.70) 3.30 (1.08)	1.55 (0.75) 3.61 (1.37)	1.57 (0.75) 3.67 (1.17)	1.68 (0.78) 3.80 (1.23)	1.62 (0.59) 3.35 (0.78)	Group**	ME < L
Housing risk index	Medium Low	1.10 (0.30) 1.87 (0.46)	1.24 (0.44) 1.85 (0.37)	1.27 (0.46) 1.74 (0.45)	1.38 (0.50) 1.83 (0.48)	1.23 (0.43) 1.68 (0.48)	1.10 (0.30) 1.65 (0.49)	Group**	ME < L
Social class of head of household	Medium Low	1.76 (0.70) 3.48 (0.67)	2.05 (0.74) 3.15 (0.37)	1.68 (0.65) 3.09 (0.67)	1.95 (0.67) 3.38 (0.58)	2.18 (0.96) 3.32 (0.56)	2.05 (0.81) 3.30 (0.70)	Group**	ME < L
**Anthropometric characteristics**									
Height	Medium Low	1,244.45 (68.90) 1,233.65 (113.75)	1,228.95 (57.56) 1,207.25 (47.86)	1,376.45 (50.39) 1,350.48 (73.55)	1,358.95 (70.25) 1,294.58 (58.81)	1,464.05 (73.39) 1,402.68 (74.51)	1,475.55 (77.30) 1,458.87 (80.55)	Group** Age** AgexSex**	ME > LP 7 < 9 < 11 9** 11*
Weight	Medium Low	28.75 (6.52) 23.11 (3.33)	27.01 (5.90) 22.88 (3.20)	38.51 (8.87) 33.01 (7.76)	36.80 (10.89) 27.66 (6.48)	45.55 (9.83) 37.88 (11.34)	46.05 (10.16) 39.43 (12.52)	Group** Age**	ME > L 7 < 9 < 11
Abdominal circumference	Medium Low	596.95 (75.78) 529.35 (24.91)	591.32 (77.95) 521.25 (35.79)	680.14 (91.13) 601.00 (89.90)	657.50 (99.76) 549.04 (72.18)	710.82 (104.60) 634.08 (97.49)	693.82 (89.44) 612.30 (98.27)	Group** Age** Sex*	ME> L 7 < 9 < 11 B > G
Head circumference	Medium Low	516.77 (14.29) 508.96 (15.22)	506.23 (12.21) 499.85 (16.42)	529.18 (9.59) 513.00 (19.80)	518.59 (20.29) 499.46 (22.55)	530.64 (12.09) 514.24 (16.08)	526.41 (15.91) 511.57 (16.77	Group** Age Sex	ME> L 7< 9 < 11 B > G

M, mean; SD, standard deviation; ME, medium; L, low; B, boy; G, girl; **p < 0.01; *p < 0.05.

### Differences in Anthropometric Variables Between Groups

Results for anthropometric characteristics were as follows: a) weight varied according to the SES, F(1,258) = 38.54, p < .001 (partial η^2^ = .130), and age, F(2,258) = 83.12, p < .001 (partial η^2^ = .392); b) height varied according to the SES, F(1,258) = 14.29, p < .001 (partial η^2^ = .052), and age, F(2,258) = 207.64, p < .001 (partial η^2^ = .617); c) the abdominal circumference varied according to the SES, F(1,258) = 62.46, p < .001 (partial η^2^ = .195), and age, F(2,258) = 34.19, p < .001 (partial η^2^ = .210); and d) the cranial circumference varied according to the SES, F(1,258) = 45.38, p < .001 (partial η^2^ = .150), and age, F(2,258) = 13.55, p < .001 (partial η^2^ = .095) (see **Table 1**). In all cases, values were lower for the children in the low-SES group than for the children in the medium-SES group. In regard to age, *post-hoc* analyses showed differences among the three age groups, with lower scores for the younger than older children. There were also differences for the main effect of the sex variable on head circumference, whose values were higher in boys than in the girls. Finally, the age x sex interaction was significant for height, with differences between boys and girls in the 9- and 11-year-old age groups.

### Differences Between Socioeconomic Status Groups in Psychopathology and Social Competence

A significant difference between SES groups were found for all syndromes gathered in the CBCL except for thought problems (**Table 2**). In comparison to the medium-SES group, the low-SES group obtained higher scores for depression, F(1,258) = 39.234, p < .001 (partial η^2^ = .132); somatic complaints, F(1,258) = 21.021, p < .001 (partial η^2^ = .075); social, F(1,258) = 35.566, p < .001 (partial η^2^ = .121); attention problems, F(1,258) = 49.792, p < .001 (partial η^2^ = .162); rule-breaking behavior, F(1,258) = 38.436, p < .001 (partial η^2^ = .130); and aggressive behavior, F(1,258) = 47.404, p < .001 (partial η^2^ = .155).

**Table 2:** Group, age, and sex differences and interaction on syndrome scales [Child Behavior Checklist (CBCL)].

Scales	Group	7 years		9 years		11 years		*p*	*Post hoc*
	Medium-SES (n = 133) Low-SES (n = 141)	Boy (n = 45) M (SD)	Girl (n = 44) M (SD)	Boy (n = 45) M (SD)	Girl (n = 46) M (SD)	Boy (n = 47) M (SD)	Girl (n = 45) M (SD)		
**CBCL**									
Anxiety	Medium Low	4.30 (3.43) 4.96 (4.25)	3.59 (3.47) 5.90 (4.23)	4.36 (3.35) 4.65 (3.10)	6.14 (4.12) 5.50 (3.19)	3.36 (3.35) 6.56 (4.80)	4.76 (2.43) 6.57 (3.88)	Group**	ME < L
Depression	Medium Low	1.83 (1.80) 2.48 (1.97)	1.59 (2.22) 3.70 (3.39)	1.68 (1.67) 3.09 (2.61)	2.45 (2.41) 4.50 (3.61)	1.50 (1.74) 5.52 (3.75)	1.81 (1.75) 3.61 (3.07)	Group** AgexSex*	ME < L 9: B < G 11: B > G
Somatic complaints	Medium Low	1.43 (1.67) 2.65 (2.99)	0.82 (1.30) 3.25 (3.57)	1.09 (1.38) 2.39 (2.62)	1.86 (1.96) 2.79 (2.84)	1.64 (2.38) 3.60 (3.91)	2.14 (2.03) 2.91 (2.52)	Group**	ME < L
Social problems	Medium Low	2.26 (1.98) 4.35 (3.54)	3.00 (2.81) 4.40 (2.98)	2.27 (1.75) 4.17 (3.23)	3.59 (2.75) 5.21 (3.16)	1.77 (2.16) 5.48 (3.45)	2.43 (1.96) 3.96 (2.95)	Group**	ME < L
Thought problems	Medium Low	1.43 (1.56) 1.39 (1.90)	1.23 (1.90) 1.15 (1.90)	1.05 (1.17) 1.30 (1.33)	1.64 (2.08) 1.75 (2.38)	1.23 (1.27) 1.64 (1.76)	0.67 (1.02) 0.82 (1.40)	AgexSex*	9: B < G 11: B > G
Attention problems	Medium Low	370 (3.25) 5.48 (3.32)	2.95 (2.08) 7.20 (4.54)	2.45 (2.37) 7.78 (5.63)	3.27 (2.73) 4.96 (5.39)	3.45 (2.02) 8.44 (5.79)	2.95 (2.89) 5.22 (3.90)	Group**	ME < L
Rule-breaking behavior	Medium Low	2.00 (1.48) 3.91 (4.37)	1.05 (1.56) 4.30 (3.64)	0.95 (1.13) 3.52 (4.08)	1.14 (1.83) 2.46 (1.82)	1.82 (1.82) 3.52 (3.58)	0.71 (0.85) 2.00 (2.32)	Group** Sex*	ME < L B > G
Aggressive behavior	Medium Low	7.04 (5.05) 9.09 (7.25)	4.68 (4.19) 9.55 (6.64)	5.23 (4.48) 11.78 (8.48)	5.00 (3.68) 8.46 (6.63)	4.09 (3.07) 10.76 (8.70)	4.33 (1.96) 10.13 (4.08)	Group**	ME < L

M, mean; SD, standard deviation; ME, medium; L, low; B, boy; G, girl; **p < 0.01; *p < 0.05.

As shown in **Table 3** (CBCL psychopathologic scales), the low-SES group obtained higher scores than the medium-SES group in internalizing problems F(1,258) = 30.757, p < .001 (partial η^2^ = .107); externalizing problems, F(1,258) = 52.174, p < .001 (partial η^2^ = .168); and total problems, F(1,258) = 61.362, p < .001 (partial η^2^ = .192).

**Table 3:** Group, age, and sex differences and interaction on internalizing, externalizing, and total problems [Child Behavior Checklist (CBCL)].

**Scales**	Group	7 years		9 years		11 years		*p*	*Post hoc*
	Medium-SES (n = 133) Low-SES (n = 141)	Boy (n = 45) M (SD)	Girl (n = 44) M (SD)	Boy (n = 45) M (SD)	Girl (n = 56) M (SD)	Boy (n = 47) M (SD)	Girl (n = 45) M (SD)		
**CBCL**									
Internalizing problems	Medium Low	7.57 (4.50) 10.09 (7.17)	6.00 (4.67) 12.85 (8.86)	7.14 (5.29) 10.13 (6.73)	10.45 (6.88) 12.79 (7.43)	6.50 (5.40) 15.68 (10.79)	8.71 (4.42) 13.09 (7.83)	Group**	ME < L
Externalizing problems	Medium Low	9.04 (5.67) 13.00 (11.10)	5.73 (5.33) 13.85 (9.55)	6.18 (5.05) 15.30 (11.92)	6.14 (4.85) 10.92 (7.99)	5.91 (4.56) 14.28 (11.64)	5.05 (3.37) 12.13 (5.18)	Group**	ME < L
Total problems	Medium Low	28.96 (15.67) 40.30 (23.47)	22.95 (16.59) 45.75 (22.60)	23.55 (12.90) 44.00 (24.94)	30.23 (16.11) 40.71 (22.62)	23.41 (12.51) 51.52 (27.61)	23.29 (9.46) 40.57 (15.27)	Group**	ME < L

M, mean; SD, standard deviation; ME, medium; L, low; B, boy; G, girl; **p < 0.01.

As shown in **Table 4** (CBCL social competence scales), the low-SES group obtained lower scores than the medium-SES group in social activities, F(1,253) = 12.114, p < .001 (partial η^2^ = .045); school, F(1,256) = 64.122, p < .001 (partial η^2^ = .200); and total social competence, F(1,249) = 35.289, p < .001 (partial η^2^ = .124).

**Table 4:** Group, age, and sex differences and interaction on Social Competence Scales [Child Behavior Checklist (CBCL)].

Scales	Group	7 years		9 years		11 years		*p*	*Post hoc*
	Medium-SES (n = 133) Low-SES (n = 141)	Boy (n = 45) M (SD)	Girl (n = 44) M (SD)	Boy (n = 45) M (SD)	Girl (n = 46) M (SD)	Boy (n = 47) M (SD)	Girl (n = 45) M (SD)		
**CBCL**									
Activities	Medium Low	5.89 (2.95) 5.39 (1.73)	7.05 (3.00) 5.37 (2.41)	7.18 (1.94) 5.30 (1.64)	5.71 (2.94) 6.57 (2.41)	7.30 (2.44) 6.00 (2.95)	6.86 (2.31) 6.04 (1.94)	Group**	ME > L
Social	Medium Low	5.26 (2.05) 4.32 (2.23)	5.23 (1.69) 3.95 (1.96)	5.62 (1.24) 4.39 (2.04)	4.82 (1.74) 4.33 (1.79)	5.30 (1.80) 4.96 (1.95)	5.57 (1.67) 5.00 (2.28)	Group**	ME > L
School	Medium Low	5.22 (0.45) 4.65 (0.93)	5.05 (0.59) 4.70 (0.86)	5.09 (0.43) 4.13 (0.87)	5.18 (0.59) 4.30 (1.11)	5.13 (0.71) 3.88 (1.05)	5.19 (0.60) 4.65 (0.65)	Group**	ME > L
Total social competence	Medium Low	16.59 (4.07) 14.68 (2.93)	17.86 (4.17) 14.16 (3.64)	18.57 (2.06) 14.17 (2.98)	16.00 (4.25) 14.95 (3.30)	17.95 (3.90) 15.13 (3.96)	17.81 (3.45) 15.87 (3.61)	Group**	ME > L
									

M, mean; SD, standard deviation; ME, medium; L, low; B, boy; G, girl; **p < 0.01.

As shown in **Table 5** (CBCL DSM-oriented Scales), the low-SES group obtained higher scores than the medium-SES group in all problems except for anxiety and obsessive-compulsive disorder (OCD) problems, as follows: affective problems, F(1,258) = 36.531, p < .001 (partial η^2^ = .124); somatic problems, F(1,258) = 14.155, p < .001 (partial η^2^ = .052); ADHD problems, F(1,258) = 26.749 (partial η^2^ = .094), p < .001; conduct problems, F(1,258) = 46.219, p < .001 (partial η^2^ = .152); sluggish cognitive tempo (SCT) problems, F(1,258) = 23.543, p < .001 (partial η^2^ = .084); and post-traumatic stress disorder (PTSD) problems, F(1,258) = 28.824, p < .001 (partial η^2^ = .100).

**Table 5:** Group, age, and sex differences and interaction on Diagnostic and Statistical Manual (DSM)-oriented scales [Child Behavior Checklist (CBCL)].

Scales	Group	7 years		9 years		11 years		*p*	*Post hoc*
	Medium-SES (n = 133) Low-SES (n = 141)	Boy (n = 45) M (SD)	Girl (n = 44) M (SD)	Boy (n = 45) M (SD)	Girl (n = 46) M (SD)	Boy (n = 47) M (SD)	Girl (n = 45) M (SD)		
**CBCL**									
Affective problems	Medium Low	1.61 (1.83) 3.17 (2.46)	2.18 (2.40) 3.85 (2.94)	1.32 (1.52) 3.52 (2.56)	2.59 (2.70) 3.75 (2.80)	1.95 (1.50) 5.04 (3.96)	1.71 (1.45) 3.13 (2.53)	Group** AgexSex*	ME < L 9: B < G 11: B > G
Anxiety problems	Medium Low	3.04 (2.48) 3.52 (2.48)	2.55 (2.32) 2.95 (2.19)	2.95 (2.26) 2.57 (2.54)	4.09 (2.81) 3.63 (2.20)	2.09 (2.09) 3.60 (2.71)	3.29 (1.79) 3.95 (2.92)		
Somatic problems	Medium Low	0.70 (0.88) 1.43 (2.27)	0.41 (0.80) 1.85 (2.13)	0.64 (0.90) 1.04 (1.64)	0.95 (1.76) 1.46 (1.91)	0.86 (1.49) 1.92 (2.52)	1.19 (1.54) 2.00 (2.49)	Group**	ME < L
ADHD problems	Medium Low	4.57 (3.98) 6.13 (3.36)	3.59 (3.11) 6.60 (4.08)	3.00 (2.20) 6.74 (4.21)	4.18 (3.35) 4.17 (3.42)	3.95 (2.50) 6.44 (4.32)	2.86 (3.31) 5.30 (3.34)	Group**	ME < L
Oppositionalproblems	Medium Low	3.09 (2.48) 3.13 (2.83)	2.23 (2.02) 3.60 (3.05)	1.91 (2.04) 4.17 (3.26)	2.00 (1.85) 2.75 (2.63)	1.82 (1.74) 3.88 (3.33)	1.29 (1.49) 3.35 (1.67)	Group**	ME < L
Conduct problems	Medium Low	1.91 (1.47) 4.65 (5.77)	1.00 (1.48) 5.05 (4.49)	0.95 (1.36) 4.74 (5.99)	0.95 (1.68) 3.08 (2.90)	1.82 (2.15) 4.44 (4.93)	0.76 (0.77) 2.83 (2.81)	Group**	ME < L
SCT	Medium Low	0.57 (1.12) 0.78 (1.41)	0.68 (0.89) 1.50 (1.88)	0.36 (0.66) 2.00 (2.49)	1.00 (1.31) 1.96 (2.49)	0.55 (0.96) 2.24 (2.37)	0.71 (0.96) 1.26 (1.60)	Group**	ME < L
OCD	Medium Low	1.61 (1.90) 1.35 (1.37)	1.23 (1.57) 1.55 (1.61)	1.77 (2.20) 1.26 (1.18)	1.95 (2.15) 1.13 (1.42)	1.14 (1.32) 2.36 (2.25)	1.43 (1.33) 1.74 (1.60)	GroupxAge*	9: ME > L 11: ME < L
PTSD	Medium Low	4.26 (2.96) 5.65 (3.82)	3.18 (2.34) 6.00 (3.28)	4.32 (3.08) 5.48 (4.52)	5.18 (3.39) 6.38 (4.57)	2.77 (1.77) 7.92 (5.26)	3.81 (2.09) 6.35 (4.14)	Group** GroupxAge*	ME < L 7: ME < L 9: ME = L 11: ME < L

M, mean; SD, standard deviation; ME, medium; L, low; B, boy; G, girl; SCT, sluggish cognitive tempo; OCD, obsessive-compulsive disorder; PTSD, post-traumatic stress disorder; **p < 0.01; *p < 0.05.

Children were classified into three groups according to their T-score in each CBCL scale following proposals of the CBCL authors (34): a) no problems/normal, T-score < 65; b) borderline, typical score of 65–69; and c) clinical problems, T-score > 69. Children were also classified into three groups according to their scores in internalizing, externalizing, and total problem scales: a) no problems/normal, T-score < 60; b) borderline, T-score of 60–63; and c) clinical problems, T-score > 63. These results are exhibited in **Tables 6**–**8**, which report on the number of children in each group, the percentage, and the between-group differences evaluated using the chi-square test. In comparison to the medium-SES group, the low-SES group had a significantly and markedly higher percentage of children with clinical problems in all scales with the exception of anxiety and thought syndromes, DSM-oriented anxiety problems and OCD scales, and a significantly higher percentage of children with clinical internalizing (21.9 *vs*. 8.3%) and externalizing (18.5 *vs*. 2.6%) problems. These values were especially high for the depression scale (40.8 *vs*. 22.3%) (**Tables 6**–**8**).

**Table 6:** Percentages of children with and without clinical problems in Syndrome scales [Child Behavior Checklist (CBCL)] and analysis of differences between medium- and low-socioeconomic status (SES) groups.

Scales	Group					
	Medium-SES (n = 133) Low-SES (n = 141)	Without problem n (%)	Borderline n (%)	Clinical n (%)	χ^2^	*p*
Anxiety	Medium Low	104 (32.9%) 97 (36.6%)	15 (5.7%) 25 (9.4%)	8 (3%) 16 (6%)	4.962	0.084
Depression	Medium Low	53 (20%) 24 (9.1%)	15 (5.7%) 6 (2.3%)	59 (22.3%) 108 (40.8%)	28.749	<0.001**
Somatic complaints	Medium Low	115 (43.4%) 103 (38.9%)	10 (3.8%) 19 (6%)	2 (0.8%) 16 (6%)	13.910	0.001**
Social problems	Medium Low	119 (44.9%) 107 (40.4%)	5 (1.9%) 16 (6%)	3 (1.1%) 15 (5.7%)	13.967	0.001**
Thought problems	Medium Low	124 (46.8%) 129 (48.7%)	2 (0.8%) 7 (2.6%)	1 (0.4%) 2 (0.8%)	2.758	0.252
Attention problems	Medium Low	122 (46%) 96 (36.2%)	5 (1.9%) 19 (7.2%)	0 (0%) 23 (8.7%)	33.869	<0.001**
Rule-breaking behavior	Medium Low	124 (46.8%) 105 (39.6%)	2 (0.8%) 12 (4.5%)	1 (0.4%) 21 (7.9%)	26.490	<0.001**
Aggressive behavior	Medium Low	118 (44.5%) 89 (33.6%)	7 (2.6%) 22 (8.3%)	2 (0.8%) 27 (10.2%)	32.973	<0.001**

**p < 0.01.

**Table 7:** Percentages of children with and without clinical problems in internalizing, externalizing, and total problem scales [Child Behavior Checklist (CBCL)] and analysis of differences between medium- and low-socioeconomic status (SES) groups.

Scales	Group					
	Medium-SES (n = 133) Low-SES (n = 141)	Without problem n (%)	Borderline n (%)	Clinical n (%)	χ^2^	*p*
Internalizing problems	Medium Low	86 (32.5%) 62 (23.4%)	19 (7.2%) 18 (6.8%)	22 (8.3%) 58 (21.9%)	19.696	<0.001**
Externalizing problems	Medium Low	106 (40%) 75 (28.3%)	14 (5.3%) 14 (5.3%)	7 (2.6%) 49 (18.5%)	36.416	<0.001**
Total problems	Medium Low	104 (32.9%) 68 (25.7%)	17 (6.4%) 22 (8.3%)	6 (2.3%) 48 (18.1%)	40.456	<0.001**

**p < 0.01.

**Table 8:** Percentages of children with and without clinical problems in Diagnostic and Statistical Manual (DSM)-oriented scales [Child Behavior Checklist (CBCL)] and analysis of differences between medium- and low-socioeconomic status (SES) groups.

Scales	Group					
	Medium-SES (n = 133) Low-SES (n = 141)	Without problem n (%)	Borderline n (%)	Clinical n (%)	χ^2^	*p*
Affective problems	Medium Low	113 (42.6%) 97 (36.6%)	11 (4.2%) 18 (6.8%)	3 (1.1%) 23 (8.7%)	17.868	<0.001**
Anxiety problems	Medium Low	87 (32.8%) 89 (33.6%)	19 (7.2%) 18 (6.8%)	21 (7.9%) 31 (11.7%)	1.519	0.468
Somatic problems	Medium Low	119 (44.9%) 111 (41.9%)	5 (1.9%) 12 (4.5%)	3 (1.1%) 15 (5.7%)	10.722	0.005**
ADHD problems	Medium Low	113 (42.6%) 94 (35.5%)	10 (3.8%) 22 (8.3%)	4 (1.5%) 22 (8.3%)	18.280	<0.001**
Oppositional problems	Medium Low	115 (43.4%) 105 (39.6%)	9 (3.4%) 8 (3%)	3 (1.1%) 25 (9.4%)	17.372	<0.001**
Conduct problems	Medium Low	123 (46.4%) 93 (35.1%)	4 (1.5%) 20 (7.5%)	0 (0%) 25 (9.4%)	39.445	<0.001**
SCT	Medium Low	119 (44.9%) 103 (38.9%)	4 (1.5%) 9 (3.4%)	4 (1.5%) 26 (9.8%)	18.785	<0.001**
OCD	Medium Low	118 (44.5%) 134 (50.6%)	3 (1.1%) 0 (0%)	6 (2.3%) 4 (1.5%)	3.966	0.138
PTSD	Medium Low	119 (44.9%) 99 (37.4%)	6 (2.3%) 25 (9.4%)	2 (0.8%) 14 (5.3%)	22.061	<0.001**

SCT, sluggish cognitive tempo; OCD, obsessive-compulsive disorder; PTSD, post-traumatic stress disorder **p < 0.01.

**Table 9** displays the results of simple linear regression models for the main CBCL variables (internalizing problems, externalizing problems, and total problems). All models showed statistically significant differences (p < .001) in all variables. The housing risk index (standardized β = 0.204, p = .005) and school competence (standardized β = −0.214, p = .001) emerged as a significant predictor of internalizing problems and school competence as a significant predictor of externalizing problems (standardized β = −0.403, p < .001) and total problems (standardized β = −0.404, p < .001).

**Table 9:** Linear regression models using score for internalizing, externalizing, and total problems as criteria and sex, age variables, socioeconomic status (SES) dimensions, and social competences as predictor.

Scales	Variables	Standardized β	*t*	*p*	Inferior 95% CI	Superior 95% CI	F model	*R^2^*	Adjusted *R^2^*
Internalizing problems							F(8, 253) = 5.962***	.163	.136
	Age	0.084	1.406	0.161	−0.156	0.937			
	Sex	0.098	1.656	0.099	−0.279	3.220			
	MLE	−0.048	−0.634	0.527	−1.111	0.570			
	HRI	0.204	2.862	0.005**	0.942	5.101			
	SCL	0.143	1.819	0.070	−0.093	2.334			
	Activities	0.002	0.026	0.979	−0.375	0.385			
	Social	0.061	0.959	0.339	−0.248	0.718			
	School	−0.214	−3.317	0.001**	−2.941	−0.750			
Externalizing problems							F(8,253) = 9.251***	0.232	0.207
	Age	−0.094	−1.647	0.101	−1.094	0.098			
	Sex	−0.054	−0.960	0.338	−2.838	0.977			
	MLE	−0.043	−0.589	0.556	−1.191	0.643			
	HRI	0.089	1.311	0.191	−0.759	3.777			
	SCL	0.116	1.542	0.124	−0.287	2.360			
	Activities	−0.025	−0.412	0.681	−0.501	0.328			
	Social	−0.008	−0.125	0.900	−0.561	0.493			
	School	−0.0403	−6.529	<0.001***	−5.157	−2.767			
Total problems							F(8,253) = 11.768***	0.278	0.254
	Age	−0.040	−0.721	0.472	−1.959	0.909			
	Sex	0.006	0.115	0.908	−4.321	4.858			
	MLE	−0.043	−0.605	0.546	−2.883	1.528			
	HRI	0.170	2.567	0.011*	1.656	12.568			
	SCL	0.153	2.092	0.037*	0.198	6.566			
	Activities	−0.017	−0.297	0.767	−1.147	0.847			
	Social	0.074	1.251	0.212	−0.463	2.073			
	School	−0.404	−6.736	<0.001***	−12.709	−6.958			

MLE, maternal level of education; HRI, Housing Risk Index; SCL, social class level of head of household, *p < 0.05, **p < 0.01, ***p < 0.001.

## Discussion

This study examined the impact of a low SES on the psychopathology of children living in a developing country, considering three age groups (7, 9, and 11 years). In comparison to children in a medium-SES environment, those in a low-SES environment had more internalizing and externalizing problems, with a higher prevalence of most syndromes studied and lower scores in social competence skills. A larger percentage of children in the low- *versus* medium-SES group had clinical problems in scales related to internalizing and externalizing problems. Finally, the housing risk index and school competence were the two main predictors of internalizing and externalizing problems in this population.

Higher scores were obtained by the low- *versus* medium-SES group in the three CBCL psychopathology scales (externalizing, internalizing, and total problems). According to the present findings, this type of problem remains present at the ages of 7, 9, and 11 years old in children with low SES, with negative effects on all psychopathological functions and an evident presence of emotional problems. It has also been suggested that malnourished children are more likely to suffer from post-traumatic stress, chronic fatigue syndrome, and depression, among other psychopathological manifestations (38).

In line with the results of previous studies, not all SES factors were associated with the emergence of emotional problems to the same degree (14, 25). The factors that best predicted the presence of emotional problems in our study were the housing risk indicator and school competence deficits. The remaining variables did not demonstrate a significant relationship, although a non-significant trend was observed for the social class of the head of the household. We highlight that the educational level of the mother was not related to the risk of emotional problems in our regression model, which may be attributable to the similarly low educational level of the mothers in both low- and medium-SES groups.

The children in the low-SES group were much more vulnerable to internalizing and externalizing clinical problems in comparison to those in the medium-SES group, and a large proportion of them suffered from clinical depressive disorders. Among previous studies on the risk of low SES for the mental health of children and adolescents, some found that the SES had a greater impact on externalizing disorders (31), whereas we found a generalized association with all problems as well as social competence.

In comparison to the medium-SES group, a greater effect size was observed for the sub-scales related to externalizing behavior in the low-SES group. Previous studies have shown that toxins, ambient noise, and neighbor quality, among many other environmental conditions, are associated with a larger number of these types of problem, including impaired impulse control and higher levels of aggression (15).

Finally, although the children under study had no previous diagnosis of psychological or cognitive impairment, the results for the low-SES group showed very high values in subscales for depression and aggressive behavior, among others. As previously reported (30), it appears to be difficult to correctly identify severe externalizing and internalizing symptoms in children, hampering their receipt of appropriate treatments or interventions.

The present data underline the need for early interventions in infancy to reduce mental health problems among children and adolescents in situations of chronic poverty, as previously proposed (39–48). These should consider multiple aspects related to the well-being of children, including performance at school and housing conditions. It is also desirable to involve parents and to adjust interventions to the reality of family life in socially disadvantaged settings. Shonkoff, Richter, van der Gaag, and Bhutta (49) reviewed interventions designed to improve the survival and development of children with low SES and concluded that the combination of nutritional interventions and psychosocial stimulation was the most widely supported approach.

Given that this was not a longitudinal study, it was not possible to rule out the effect of other variables on the differences observed among the three age groups. Future longitudinal studies are needed to control the effect of the exposure to low-SES, as well as other central variables regarding the SES such as physical conditions at home, family income, and the perceived level of stress. A further limitation was the inability to differentiate among the effects on socio-emotional development of specific aspects of low-SES (e.g., poverty, malnutrition, infant abuse, etc.). However, we contribute the first report of this nature in a population of Ecuadorian children, and the results will help in the development of programs to screen for mental health problems in children in disadvantaged settings and to detect indicators of cognitive, emotional, and social vulnerability.

Investigation of the different factors underlying the relationship between a disadvantaged environment and mental health is beyond the scope of our study. Various hypotheses have been developed, including the benefits for learning of a more stimulating and protected environment (50) and the negative effects of a low SES on the development of brain circuits and metabolism-regulating systems, increasing the likelihood of long-term problems in learning, behavior, mental, and physical health (51). It has been observed that the experience of multiple social and economic stressors generates emotional problems related to fear and anxiety in young people, increasing disruptive behavior and alterations in executive function and self-regulation (52). Further research is warranted to elucidate these issues.

## Conclusion

In conclusion, schoolchildren with low SES in a developing country had more emotional disorders, including externalizing and internalizing problems, in comparison to those with medium SES. Housing risk index and school competence emerged as the main predictors of the children's CBCL scores. These findings support the need for short-term and long-term preventive programs to counter the negative effects of social deprivation. Future research is required on emotional variables in children with low SES and on related aspects, including the influence of genetics and the role of specific brain mechanisms. It is of particular interest to determine whether the trend to a greater impact of low SES at higher age (11 *vs*. 7 years of age) detected in this study continues up to adulthood.

## Data Availability Statement

The datasets generated for this study are available on request to the corresponding authors.

## Ethics Statement

Written consent was obtained from the parents/guardians of the children for their participation in the study, which was approved by the ethical committee of the local University (Ref: A3/042954/11).

## Author Contributions

MNP-M, CB-G, FC-Q, and MP-G designed the tasks and collected the data. MNP-M, MF-A, AF, and CB-G undertook the statistical analysis, and MNP-M wrote the first draft of the manuscript. All authors contributed to and have approved the final manuscript.

## Funding

This study was supported by the Spanish Agency for International Development Cooperation (AECID) [A3/042954/11] (PI: FC-Q) and Conselleria d'Educació, Investigació, Cultura I Esport de la Generalitat Valenciana (R+D+i projects developed by emerging research groups) [GV/2017/166] (PI: MF-A).

## Conflict of Interest

The authors declare that the research was conducted in the absence of any commercial or financial relationships that could be construed as a potential conflict of interest.
